# Temporal trends in stroke incidence in South Asian, Chinese and white patients: A population based analysis

**DOI:** 10.1371/journal.pone.0175556

**Published:** 2017-05-18

**Authors:** Nadia A. Khan, Finlay A. McAlister, Louise Pilote, Anita Palepu, Hude Quan, Michael D. Hill, Jiming Fang, Moira K. Kapral

**Affiliations:** 1 Division of General Internal Medicine, University of British Columbia, Vancouver, British Columbia, Canada; 2 Center for Health Evaluation and Outcomes Sciences, Vancouver, British Columbia, Canada; 3 Division of General Internal Medicine, University of Alberta, Edmonton, Alberta, Canada; 4 Patient Health Outcomes Research and Clinical Effectiveness Unit, University of Alberta, Edmonton, Alberta, Canada; 5 Department of Medicine, Division of Clinical Epidemiology, The Research Institute of the McGill University Health Center, Montréal, Québec, Canada; 6 Department of Medicine, Division of General Internal Medicine, McGill University, Montréal, Québec, Canada; 7 Department of Community Health Sciences, University of Calgary, Calgary, Alberta, Canada; 8 Department of Clinical Neurosciences, University of Calgary, Calgary, Alberta, Canada; 9 Institute for Clinical Evaluative Sciences, Toronto, Ontario, Canada; 10 Department of Medicine, Division of General Internal Medicine, University of Toronto, Toronto, Ontario, Canada; Shanghai Institute of Hypertension, CHINA

## Abstract

**Background:**

Little is known about potential ethnic differences in stroke incidence. We compared incidence and time trends of ischemic stroke and primary intracerebral hemorrhage in South Asian, Chinese and white persons in a population-based study.

**Methods:**

Population based census and administrative data analysis in the provinces of Ontario and British Columbia, Canada using validated ICD 9/ICD 10 coding for acute ischemic and hemorrhagic stroke (1997–2010).

**Results:**

There were 3290 South Asians, 4444 Chinese and 160944 white patients with acute ischemic stroke and 535 South Asian, 1376 Chinese and 21842 white patients with intracerebral hemorrhage. South Asians were younger than whites at onset of stroke (70 vs. 74 years for ischemic and 67 vs. 71 years for hemorrhagic stroke). Age and sex adjusted ischemic stroke incidence in 2010 was 43% lower in Chinese and 63% lower in South Asian than in White patients. Age and sex adjusted intracerebral hemorrhage incidence was 18% higher in Chinese patients, and 66% lower in South Asian relative to white patients. Stroke incidence declined in all ethnic groups (relative reduction 69% in South Asians, 25% in Chinese, and 34% in white patients for ischemic stroke and for intracerebral hemorrhage, 79% for South Asians, 51% for Chinese and 30% in white patients).

**Conclusion:**

Although stroke rates declined across all ethnic groups, these rates differed significantly by ethnicity. Further study is needed to understand mechanisms underlying the higher ischemic stroke incidence in white patients and intracerebral hemorrhage in Chinese patients.

## Introduction

Rates of acute myocardial infarction vary substantially across ethnic groups. Although stroke is a leading cause of death world wide, less is known on whether there are ethnic variations in stroke incidence [1]. Both ischemic and primary intracerebral hemorrhage are increasing in low to middle income populations in parallel with their rising prevalence of stroke risk factors including diabetes, hypertension and atrial fibrillation [2–4]. While Western countries experienced a 42% decrease in stroke incidence over the past 20 years, India and China reported substantial increases in stroke incidence over the same time period, with higher reported rates of intracerebral hemorrhage than in North America [1, 5]. Migrants from India and China to Western countries also have higher rates of stroke risk factors, including hypertension, than the general population [6,7]. Chinese and South Asian women living in Canada also have a higher proportionate mortality for stroke than European descent women [8]. These population estimates raise significant concern that Asian populations may be at increased risk of stroke and higher rates of intracerebral hemorrhage. Comparing population based stroke rates between countries is challenging given differences in data collection, access to health care, and brain imaging [4]. Moreover, there are little existing data distinguishing hemorrhagic from ischemic stroke rates in these populations despite the fact that risk factors and prognosis are distinct between stroke types.

Better estimates of stroke incidence by ethnicity are needed to help prioritize health system funding and research efforts for vulnerable populations. We sought to compare the incidence of ischemic stroke and primary intracerebral hemorrhage in South Asian (originating from Pakistan, India or Bangladesh), Chinese (originating from China, Taiwan or Hong Kong), and non-Chinese, non-South Asian (predominantly European descent) women and men in a population-based study under a universal access health care system in Ontario and British Columbia, Canada.

## Materials and methods

### Setting and data sources

We used administrative data from British Columbia (BC) (1999–2009) and Ontario (1997–2010) in Canada. BC and Ontario are ethnically diverse provinces with 53% of all Chinese and 83% of all South Asian persons residing in these two provinces [9]. We used hospital discharge abstracts data that include all inpatient services for all hospitals using International Classification of Diseases, 9th Revision (ICD-9) and 10^th^ Revision (ICD-10) codes. Demographic and surname data were obtained from provincial health insurance registration databases and medication data were obtained using the BC PharmaNet and Ontario Drug Databases. Population census data were used to determine population counts for each ethnic group [9]. We obtained de-identified linked health datasets through the Institute for Clinical Evaluative Sciences in Ontario and Population Data BC with approval of relevant data stewards and from the Research Ethics Boards of the University of Toronto and the University of British Columbia [10]. All inferences, opinions, and conclusions drawn in this report are those of the authors, and do not reflect the opinions or policies of the data stewards, Ministries of Health of BC or ON, the Institute for Clinical Evaluative Sciences, or Population Data BC.

### Study population

Data were available for all residents aged 25 years and older in British Columbia and those aged 35 years and older in Ontario with a hospitalization for acute ischemic stroke and acute hemorrhagic stroke. Stroke hospitalizations were defined using well-validated ICD-9 or ICD-10 codes (ischemic stroke ICD-9 434, 436 or ICD-10-CA I63, I64; intracerebral hemorrhage ICD-9 431 or ICD-10 I61.x) [11]. There were insufficient numbers of patients with subarachnoid hemorrhage to analyze by ethnicity.

### Categorizing ethnic group

We uses validated surname analysis to categorize patients as South Asian (from Pakistan, India or Bangladesh) or Chinese (from China, Taiwan or Hong Kong) [12,13]. The details on the validation of surname analysis are described elsewhere [14]. Data on other ethnic groups were not available (First Nations, Hispanic, Filipino, African origin).

### Stroke incidence

To determine incident cases, we identified all patients hospitalized for a most responsible diagnosis of acute ischemic stroke or primary intracerebral hemorrhage. Only the first encounter (index diagnosis) when an individual first met the stroke case definition algorithm in the study period was included (excluding subsequent encounters).

### Stroke risk factors

Comorbid conditions associated with stroke including hypertension, diabetes, atrial fibrillation, congestive heart failure, and prior cerebrovascular disease were defined using validated ICD9, ICD10 codes identified during index hospitalization [15,16]. Socioeconomic status (SES) quintile was determined using area level median income based on census data using the Statistics Canada Postal Code Conversion file.

### Statistical analysis

We used Chi square and oneway-ANOVA test where appropriate to compare baseline characteristics among ethnic groups. The yearly incidence of hospitalized stroke (per 100,000 population) was calculated using new cases of stroke for each group as the numerator and the corresponding population count as the denominator using the most recent census data. We presented the age and sex adjusted incidence in three-year intervals beginning in 1998. We determined both age and sex standardized incidence for each ethnic group as well age-standardized incidence for each ethnic and sex group for both acute ischemic stroke and intracerebral hemorrhage. Testing for time trends in standardized rates was conducted by computing Kendall’s tau-b correlation coefficient and associated P value. Trend differences by ethnic group were examined using linear regression including year and ethnic interaction terms as predictors. All analyses were conducted with SAS version 9.4 and a p-value of <0.05 was considered statistically significant.

## Results

After exclusions, there were 168,678 patients with acute ischemic stroke and 23,753 patients with primary intracerebral hemorrhagic stroke. Of all patients with ischemic stroke, 2.0% (n = 3290) were South Asian, 2.6% [n = 4444] were Chinese and 95.4% (n = 160,944) were white. Among those with primary intracerebral hemorrhage, 2.3% were South Asian (n = 535), 5.8% were Chinese (n = 1376) and 92.0% were white (n = 21842).

### Baseline characteristics and risk factors

Compared to Chinese and white patients, South Asian patients were younger at the time of both ischemic stroke and intracerebral hemorrhage (Table 1). Stroke risk factors also differed by ethnicity. In those with ischemic stroke, Chinese patients were more likely to have atrial fibrillation whereas South Asians were more likely to have diabetes compared to other ethnic groups. In those with primary intracerebral hemorrhage, white patients were less likely to have hypertension and South Asian patients were more likely to have diabetes compared with the other groups.

**Table 1:** Baseline characteristics by ethnicity.

Characteristic	Chinese	White	South Asian	P-Value
**Acute Ischemic Stroke**				
	**N = 4444**	**N = 160944**	**N = 3290**	
Age mean [SD] years Median, [interquartile range]	73.87±12.51 76[67–83]	74.48±12.59 77[67–84]	70.04±12.97 71[62–80]	<0.0001
Women	2312 [52.0%]	81869 [50.9%]	1506 [45.8%]	<0.0001
SES Quintile				
Q1 [lowest]	1186 [26.7%]	37615 [23.4%]	925 [28.1%]	<0.0001
Q2	1064 [23.9%]	34905 [21.7%]	843 [25.6%]	
Q3	805 [18.1%]	31094 [19.3%]	706 [21.5%]	
Q4	726 [16.3%]	28391 [17.6%]	457 [13.9%]	
Q5 [highest]	621 [14.0%]	27453 [17.1%]	340 [10.3%]	
Hypertension	1015 [22.8%]	31135 [19.3%]	803 [24.4%]	<0.0001
Atrial Fibrillation	518 [11.7%]	17270 [10.7%]	232 [7.1%]	<0.0001
Diabetes	796 [17.9%]	22589 [14.0%]	756 [23.0%]	<0.0001
Heart Failure	171 [3.8%]	8916 [5.5%]	183 [5.6%]	<0.0001
CVD Prior Cerebrovascular Disease	607 [13.7%]	16505 [10.3%]	361 [11.0%]	<0.0001
**Patients aged ≥65 years**	**N = 3459**	**N = 126533**	**N = 2202**	
Any antihypertensive medication within past 6 months	2386 [69.0%]	88977 [70.3%]	1531 [69.5%]	0.18
Any statin therapy within past 6 months	731 [21.1%]	28970 [22.9%]	627 [28.5%]	<0.0001
Any antiplatelet use within past 6 months	539 [15.6%]	21868 [17.3%]	423 [19.2%]	0.002
Any oral hypoglycemic agent use within past 6 months	550 [25.2%]	21610 [21.0%]	493 [37.0%]	<0.0001
**Primary Intracerebral Hemorrhage**				
	**N = 1376**	**N = 21842**	**N = 535**	
Age mean [SD] years Median, [interquartile range]	70.19±14.36 73[61–81]	71.8±13.91 75[64–82]	66.93±15.06 69[57–78]	<0.0001
Women	662 [48.1%]	10646 [48.7%]	245 [45.8%]	0.37
SES Quintile				
Q1 [lowest]	359 [26.1%]	4755 [21.8%]	149 [27.9%]	<0.0001
Q2	322 [23.4%]	4650 [21.3%]	145 [27.1%]	
Q3	251 [18.2%]	4185 [19.2%]	110 [20.6%]	
Q4	255 [18.5%]	4010 [18.4%]	69 [12.9%]	
Q5 [highest]	181 [13.2%]	4048 [18.5%]	60 [11.2%]	
Hypertension	470 [34.2%]	6248 [28.6%]	200 [37.4%]	<0.0001
Atrial Fibrillation	56 [4.1%]	1218 [5.6%]	11 [2.1%]	0.0001
Diabetes	165 [12.0%]	1920 [8.8%]	87 [16.3%]	<0.0001
Heart Failure	29 [2.1%]	615 [2.8%]	8 [1.5%]	0.06
Prior Cerebrovascular Disease	170 [12.4%]	1735 [7.9%]	37 [6.9%]	<0.0001
**Patients aged ≥65 years**	**N = 936**	**N = 15770**	**N = 317**	
Any antihypertensive medication within past 6 months	550 [58.8%]	9448 [59.9%]	185 [58.4%]	0.68
Any statin therapy within past 6 months	202 [21.6%]	3492 [22.1%]	83 [26.2%]	0.21
Any antiplatelet use within past 6 months	101 [10.8%]	2102 [13.3%]	48 [15.1%]	0.05
Any oral hypoglycemic agent use within past 6 months	116 [20.5%]	1807 [14.0%]	60 [29.9%]	<0.0001

### Incidence

**Fig 1:**
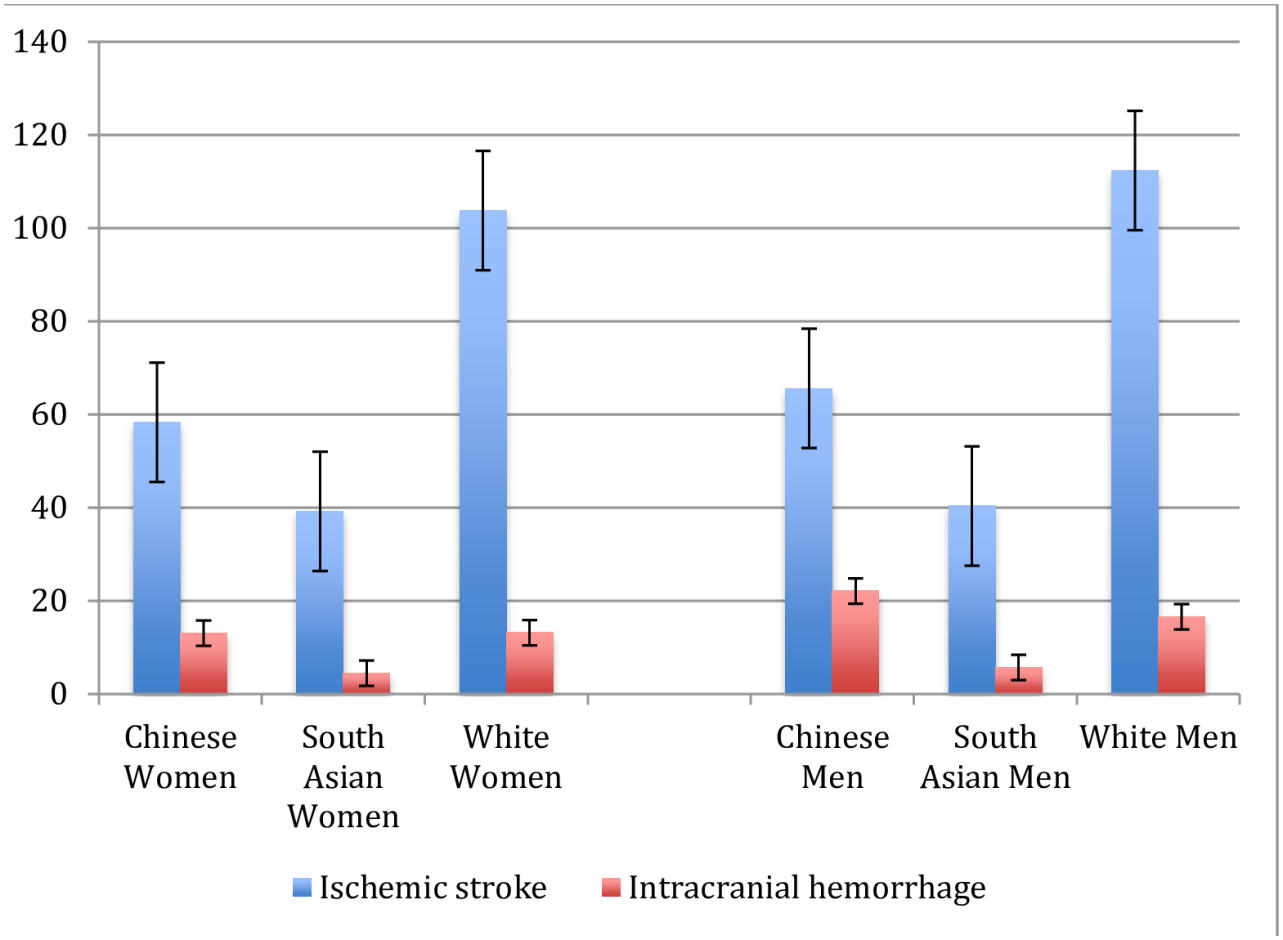
Age standardized stroke incidence with standard error by ethnicity and sex/ 100,000 persons, 2010.

**Fig 2:**
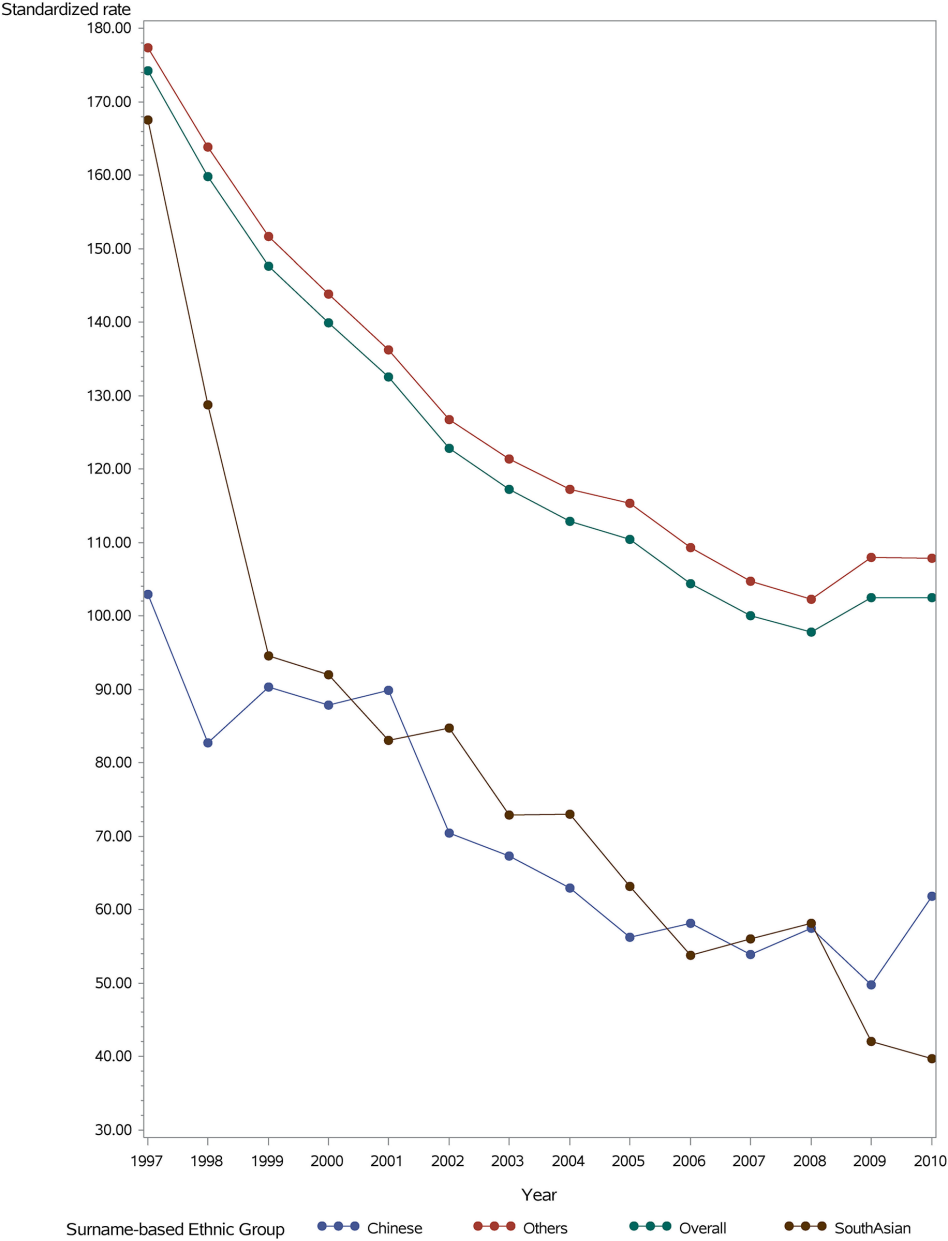
Age and sex standardized incidence of ischemic stroke per 100,000 persons by ethnic group, 1998 to 2010.

Stroke incidence varied by ethnic group (Fig 1 that depicts ethnic differences in 2010 and Fig 2 that depicts rates 1997–2010). Chinese persons had a substantially lower incidence of ischemic stroke, followed by South Asian and white patients that had higher rates of ischemic stroke. For primary intracerebral hemorrhage, stroke rates were highest among Chinese and generally similar between South Asian and white patients. The incidence of both age standardized ischemic stroke and intracerebral hemorrhage was generally higher in men than women across ethnic groups.

### Time trends

**Fig 3:**
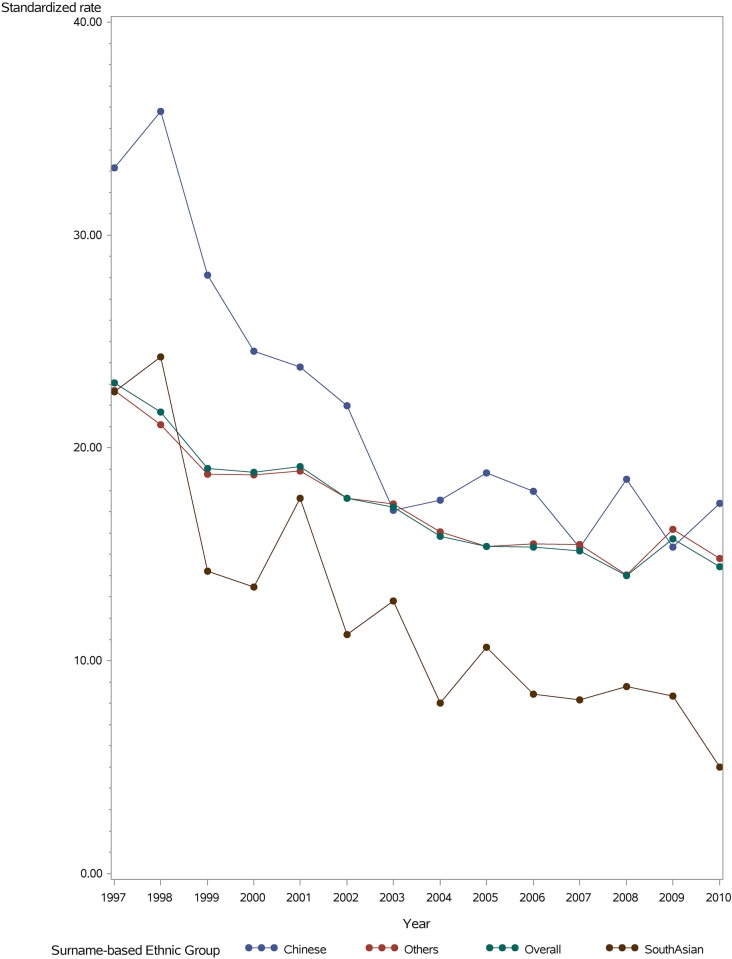
Age and sex standardized incidence of intracerebral hemorrhage per 100,000 persons by ethnic group, 1998 to 2010.

The age-/sex-standardized incidence of ischemic stroke declined between 1998 and 2010 in all ethnic groups, from 82.7 to 61.8 per 100,000 Chinese population, 128.8 to 39.7 per 100,000 South Asian population, and 163.9 to 107.9 per 100,000 white population (Fig 2) (Trend test: p <0.001 for each ethnic group; Trend difference: p = 0.0027). The relative reduction was 25% in Chinese, 69% in South Asians, and 34% in white patients for ischemic stroke. The age-/sex-standardized incidence of primary intracerebral hemorrhage also declined in all groups between 1998 and 2010, from 35.8 to 17.4 per 100,000 Chinese population, 24.3 to 5.0 per 100,000 South Asian population and 21.1 to 14.8 per 100,000 white population (Fig 3) (Trend test: p<0.001; Trend difference; p = 0.0045). The relative reduction was 51% for Chinese, 79% for South Asians and 30% in white patients.

## Discussion

We found significant variations in stroke incidence among people of different ethnic backgrounds living in Canada, with white patients having the highest incidence of ischemic stroke and those of Chinese descent having the highest incidence of primary intracerebral hemorrhage compared with other groups. We also found significant temporal declines in the incidence of both ischemic stroke and intracerebral hemorrhage among all ethnic groups and most notably for South Asians.

Ischemic stroke in this study was more common in white followed by South Asian groups and lowest among Chinese living in Canada after adjusting for age and sex. Although our stroke rates in white persons were consistent with general population rates reported in other Canadian and US data [17, 18], few studies directly compared Chinese and South Asian patients. Ischemic stroke rates in South Asian and white patients were found to be similar in a population based study in Scotland [19]. A US study did report lower rates of ischemic stroke in Asian patients however this analysis did not distinguish South Asian and Chinese [17]. The rates of stroke for South Asian Canadians were much lower compared with stroke rates reported in India [4]. Similarly, stroke rates among Chinese Canadians were lower than those living in China, although rates of overall stroke in China vary considerably (33-553/100,000) depending on geographic region [20,21].

For primary intracerebral hemorrhage, we found Chinese had a higher incidence compared with other groups. This finding is similar to a US study that reported 25–30 cases of intracerebral hemorrhage/100,000 in Chinese with a lower incidence of intracerebral hemorrhage in white patients (19-20/100,000) [17]. The international MONICA–WHO study also reported a higher rate of intracerebral hemorrhage in China compared with other countries [22].

The reasons underlying the higher risk of ischemic stroke in white and intracerebral hemorrhage in Chinese are not clear, but may be related to differences in the prevalence of vascular risk factors. White patients have higher prevalence of some stroke risk factors including obesity and smoking compared with Chinese or South Asian patients that might account for their higher risk of ischemic stroke [23, 24]. Further, as seen in the INTERSTROKE study, there may be significant variations in the magnitude of the association of these individual risk factors [25]. For example, the population attributable risk for atrial fibrillation causing stroke in South Asia was 3.1% whereas it was 17.1% in North America and Western Europe. Different ethnic groups may also have differing control of risk factors although a Canadian physical measures survey identified no differences in blood pressure control rates between ethnic groups [26]. Genetic polymorphisms for ischemic stroke, however, appear to be similar in white and South Asian persons [27]. Persons living in China also have been found to have increasing rates of hypertension over the past two decades [20]. Chinese patients were also more susceptible to microbleeds and intracranial hemorrhage from anticoagulation compared with other ethnic groups [28]. There may also be a potentially higher prevalence of cerebral amyloid angiopathy, adult onset moyamoya disease and thereby increasing risk of microbleeding in Chinese patients [29].

Our finding of a temporal decline in the incidence of both ischemic stroke and intracerebral hemorrhage in South Asian and Chinese ethnic groups pose a stark contrast to the increasing incidence of stroke in India and China [1,2]. The increasing stroke rates in developing countries may reflect increased diagnostic rates or access to hospital care, or lower access to preventative care in some regions [30]. Several studies found that the prevalence of stroke risk factors including hypertension, diabetes, smoking, alcohol consumption and atrial fibrillation increased over the past two to three decades in India and China [4,20]. Other risk factors such as poor ambient air quality may increase the risk of stroke in China and India. While stroke risk factors are also prevalent in ethnic groups in Canada [6,7], control rates of hypertension overall, the largest attributable risk factor for stroke, substantially improved in Canada over the past two decades [31]. The generally lower rates of ischemic stroke among migrants compared with those of their country of origin may also reflect a healthy immigrant effect [23].

These findings stress the importance of increasing efforts in prevention of acute stroke across populations. Although rates of acute stroke are declining, white and Chinese populations still have high rates of ischemic and hemorrhagic stroke respectively. These findings suggest increased need for primary care to screen and control stroke risk factors, in particular hypertension, and to tailor health education, and community programs to target high-risk populations. Further, the divergent rates between Chinese and South Asian Canadians compared with reports from China and India raise the possibility that health system differences may account for some of the high rates of stroke observed now in China and India. Public health policies that increase primary care screening for stroke risk factors and availability of affordable medications to treat common risk factors in underserved regions of China and India may help to alleviate the burden of stroke in these countries.

This study is the largest population based analysis of ethnic differences in stroke incidence that directly compares South Asian, Chinese and white patients living in a western country with a universal health care system. However, there are several limitations. We were unable to use self-reported ethnicity, the gold standard for determining ethnicity. Nevertheless, the surname algorithms have moderate to high accuracy compared with self-report. This study only included patients who were hospitalized for stroke. Out of hospital stroke, including fatal stroke, is reported to miss 10% of all strokes, thus our estimates are likely conservative [32]. We were unable to verify subtype of stroke or imaging modality for hemorrhagic or ischemic stroke cases. We were also unable to assess all stroke risk factors including body mass index, tobacco and alcohol use, however, we were able to determine prevalence of hypertension, statin use and prior vascular history. Finally, we were also unable to stratify this analysis into smaller ethnic subgroups recognizing there is some heterogeneity within ethnic groups.

## Conclusion

Among people living in Canada, stroke incidence differs substantially by ethnicity with those of Chinese descent experiencing significantly higher incidence of intracerebral hemorrhage and whites experiencing higher incidence of ischemic stroke compared with others. The incidence of stroke has declined between 1998 and 2010 across all ethnic groups. Further study is needed to understand the underlying causes of these observed differences in stroke incidence among ethnic groups and the variation in rates of decline in stroke, with the goal of developing targeted and perhaps personalized interventions that address the mechanisms that uniquely contribute to each ethnic group’s stroke risk

## References

[1] GBD 2013 Mortality and Causes of Death Collaborators. Global, regional, and national age-sex specific all-cause and cause-specific mortality for 240 causes of death, 1990–2013: a systematic analysis for the Global Burden of Disease Study 2013. Lancet. 2015;385[9963]:117–71. 10.1016/S0140-6736(14)61682-2 25530442 PMC4340604

[2] Zhou Z, Hu D. An epidemiological study on the prevalence of atrial fibrillation in the Chinese population of mainland China. J Epidemiol. 2008;18[5]:209–16. 10.2188/jea.JE2008021 18776706 PMC4771592

[3] Gao Y, Chen G, Tian H, Lin L, Lu J, Weng J, et al; China National Diabetes and Metabolic Disorders Study Group. Prevalence of hypertension in china: a cross-sectional study. PLoS One. 2013 6 11;8[6]:e65938 10.1371/journal.pone.0065938 23776574 PMC3679057

[4] Gunarathne A, Patel JV, Gammon B, Gill PS, Hughes EA, Lip GY. Ischemic stroke in South Asians: a review of the epidemiology, pathophysiology, and ethnicity-related clinical features. Stroke. 2009;40[6]:e415–23. 10.1161/STROKEAHA.108.535724 19390072

[5] Li B, Lou Y, Gu H, Long X, Wang T, Wei J, et al Trends in Incidence of Stroke and Transition of Stroke Subtypes in Rural Tianjin China: A Population-Based Study from 1992 to 2012. PLoS One. 2015;10[10]:e0139461 10.1371/journal.pone.0139461 26426803 PMC4591354

[6] Khan NA, Wang H, Anand S, Jin Y, Campbell NR, Pilote L, et al Ethnicity and sex affect diabetes incidence and outcomes. Diabetes Care. 2011;34[1]:96–101. 10.2337/dc10-0865 20978094 PMC3005449

[7] Quan H, Chen G, Walker RL, Wielgosz A, Dai S, Tu K, et al; Hypertension Outcome and Surveillance Team. Incidence, cardiovascular complications and mortality of hypertension by sex and ethnicity. Heart. 2013; 99[10]:715–21. 10.1136/heartjnl-2012-303152 23403406

[8] Sheth T, Nair C, Nargundkar M, Anand S, Yusuf S. Cardiovascular and cancer mortality among Canadians of European, south Asian and Chinese origin from 1979 to 1993: an analysis of 1.2 million deaths. CMAJ. 1999;161[2]:132–8. Erratum in: CMAJ 1999;161[5]:489. 10439820 PMC1230461

[9] Statsitics Canada. 2006 Web Page. http://www.statscan.ca

[10] BC Ministry of Health Data Stewardship Committee, Consolidation File [MSP Registration & Premium Billing]; Discharge Abstracts Database [Hospital Separations] and Medical Services Plan [MSP] Payment Information File; PharmaNet File; Population Data BC; BC Ministry of Health; 2012 2014. http://www.popdata.bc.ca/data

[11] Kokatailo RA, Hill MD. Coding of stroke and stroke risk factors using international classification of diseases, revisions 9 and 10. Stroke. 2005;36[8]:1776–81. 10.1161/01.STR.0000174293.17959.a1 16020772

[12] Quan H, Ghali WA, Dean S, Norris C, Galbraith PD, Faris P, et al Development and validation of a surname list to define Chinese ethnicity. Medical Care. 2006;44[4]:328 10.1097/01.mlr.0000204010.81331.a9 16565633

[13] Cummins C, Winter H, Cheng KK, Maric R, Silcocks P, Varghese C. An assessment of the Nam Pehchan computer program for the identification of names of south Asian ethnic origin. J Public Health Med. 1999;21[4]:401–6. 11469361 10.1093/pubmed/21.4.401

[14] Khan NA, Wang H, Anand S, Jin Y, Campbell NR, Pilote L, et al Ethnicity and sex affect diabetes incidence and outcomes. Diabetes Care. 2011;34[1]:96–101. 10.2337/dc10-0865 20978094 PMC3005449

[15] Hux JE, Ivis F, Flintoft V, Bica A. Diabetes in Ontario: determination of prevalence and incidence using a validated administrative data algorithm. Diabetes Care. 2002;25[3]:512–6. 11874939 10.2337/diacare.25.3.512

[16] Quan H, Khan N, Hemmelgarn BR, Tu K, Chen G, Campbell N, et al; Hypertension Outcome and Surveillance Team of the Canadian Hypertension Education Programs. Validation of a case definition to define hypertension using administrative data. Hypertension. 2009;54[6]:1423–8. 10.1161/HYPERTENSIONAHA.109.139279 19858407

[17] Nguyen-Huynh MN, Johnston SC. Regional variation in hospitalization for stroke among Asians/Pacific Islanders in the United States: a nationwide retrospective cohort study. BMC Neurol. 2005;5:21 10.1186/1471-2377-5-21 16280090 PMC1308818

[18] Tu JV, Chu A, Rezai MR, Guo H, Maclagan LC, Austin PC, et al The Incidence of Major Cardiovascular Events in Immigrants to Ontario, Canada: The CANHEART Immigrant Study. Circulation. 2015. pii: CIRCULATIONAHA.115.015345. 10.1161/CIRCULATIONAHA.115.015345 PMC4606988 26324719

[19] Bhopal RS, Bansal N, Fischbacher CM, Brown H, Capewell S; Scottish Health and Ethnic Linkage Study. Ethnic variations in the incidence and mortality of stroke in the Scottish Health and Ethnicity Linkage Study of 4.65 million people. Eur J Prev Cardiol. 2012;19[6]:1503–8. 10.1177/1741826711423217 21933831

[20] Liu M, Wu B, Wang WZ, Lee LM, Zhang SH, Kong LZ. Stroke in China: epidemiology, prevention, and management strategies. Lancet Neurol. 2007;6[5]:456–64. 10.1016/S1474-4422(07)70004-2 17434100

[21] Li B, Lou Y, Gu H, Long X, Wang T, Wei J, et al Trends in incidence of stroke and transition of stroke subtypes in rural Tianjin China: A Population-Based Study from 1992 to 2012. PLoS One. 2015;10[10]:e0139461 10.1371/journal.pone.0139461 26426803 PMC4591354

[22] Wu ZS, Yao CH, Zhao D, Wu G, Wang W, Liu J et al Sino-MONICA project A collaborative study on trends and determinants in cardiovascular diseases in China, part I: morbidity and mortality monitoring. *Circulation* 2001; 103: 462–68. 11157701 10.1161/01.cir.103.3.462

[23] Chiu M, Austin PC, Manuel DG, Tu JV. Comparison of cardiovascular risk profiles among ethnic groups using population health surveys between 1996 and 2007. CMAJ. 2010;182[8]:E301–10. 10.1503/cmaj.091676 20403888 PMC2871219

[24] Liu R, So L, Mohan S, Khan N, King K, Quan H. Cardiovascular risk factors in ethnic populations within Canada: results from national cross-sectional surveys. Open Med. 2010;4[3]:e143–53. 21687334 PMC3090103

[25] O'Donnell MJ, Chin SL, Rangarajan S, Xavier D, Liu L, Zhang H, et al; INTERSTROKE investigators. Global and regional effects of potentially modifiable risk factors associated with acute stroke in 32 countries [INTERSTROKE]: a case-control study. Lancet. 2016;388[10046]:761–75. 10.1016/S0140-6736(16)30506-2 27431356

[26] Leenen FH, Dumais J, McInnis NH, Turton P, Stratychuk L, Nemeth K, et al Results of the Ontario survey on the prevalence and control of hypertension. CMAJ. 2008;178[11]:1441–9. 10.1503/cmaj.071340 18490640 PMC2374854

[27] Yadav S, Hasan N, Marjot T, Khan MS, Prasad K, Bentley P, et al Detailed analysis of gene polymorphisms associated with ischemic stroke in South Asians. PLoS One. 2013;8[3]:e57305 10.1371/journal.pone.0057305 23505425 PMC3591429

[28] Hori M, Connolly SJ, Zhu J, Liu LS, Lau CP, Pais P, et al; RE-LY Investigators. Dabigatran versus warfarin: effects on ischemic and hemorrhagic strokes and bleeding in Asians and non-Asians with atrial fibrillation. Stroke. 2013;44[7]:1891–6. 10.1161/STROKEAHA.113.000990 23743976

[29] Mok V, Srikanth V, Xiong Y, Phan TG, Moran C, Chu S, et al Race-ethnicity and cerebral small vessel disease—comparison between Chinese and White populations. Int J Stroke. 2014;9 Suppl A100:36–42. 24661839 10.1111/ijs.12270

[30] Wasay M, Khatri IA, Kaul S. Stroke in South Asian countries. Nat Rev Neurol. 2014;10[3]:135–43. 10.1038/nrneurol.2014.13 24514866

[31] Joffres M, Falaschetti E, Gillespie C, Robitaille C, Loustalot F, et al Hypertension prevalence, awareness, treatment and control in national surveys from England, the USA and Canada, and correlation with stroke and ischaemic heart disease mortality: a cross-sectional study. BMJ Open. 2013;3[8]:e003423 10.1136/bmjopen-2013-003423 23996822 PMC3758966

[32] Williams GR: Incidence and characteristics of total stroke in the United States. BMC Neurology 2001, 1:2 10.1186/1471-2377-1-2 11446903 PMC32314

